# *Persicaria minor* (Huds.) Opiz Prevents *In Vitro* Atherogenesis by Attenuating Tumor Necrosis Factor-α-Induced Monocyte Adhesion to Human Umbilical Vein Endothelial Cells

**DOI:** 10.3390/life12101462

**Published:** 2022-09-20

**Authors:** Adila A. Hamid, Amilia Aminuddin, Nur Najmi Mohamad Anuar, Nur Izzati Mansor, Mohd Faizal Ahmad, Mohammed S. M. Saleh, Mohd Helmy Mokhtar, Azizah Ugusman

**Affiliations:** 1 Department of Physiology, Faculty of Medicine, Universiti Kebangsaan Malaysia, Kuala Lumpur 56000, Malaysia; 2 Programme of Biomedical Science, Centre for Toxicology and Health Risk Studies, Faculty of Health Sciences, Universiti Kebangsaan Malaysia, Kuala Lumpur 50300, Malaysia; 3 Department of Obstetrics and Gynaecology, Faculty of Medicine, Universiti Kebangsaan Malaysia, Kuala Lumpur 56000, Malaysia; 4 Department of Pharmacology, Faculty of Medicine, Universiti Kebangsaan Malaysia, Kuala Lumpur 56000, Malaysia

**Keywords:** endothelial cell, inflammation, monocyte adhesion, *Persicaria minor*, *Polygonum minus*

## Abstract

*Persicaria minor* (Huds.) Opiz is an herb with anti-inflammatory, antioxidant, and anti-atherosclerosis effects. Nevertheless, the mechanism underlying its anti-atherosclerosis effect is poorly comprehended. This *in vitro* study assessed the protective effects of standardized aqueous extract of *P. minor* leaves (PM) on tumor necrosis factor-α (TNF-α)-induced monocyte adhesion to human umbilical vein endothelial cells (HUVEC), which is one of the pivotal early steps in atherogenesis. The results showed that PM decreased the mRNA and protein expression of cellular adhesion molecules, vascular adhesion molecule-1 and intercellular adhesion molecule-1, resulting in reduced adhesion of monocytes to HUVEC. Additionally, PM inhibited nuclear factor kappaB (NF-κB) activation as indicated by reduced NF-κB p65 levels in TNF-α-induced HUVEC. Overall, PM could prevent *in vitro* atherogenesis by inhibiting NF-κB activation and adhesion of monocytes to HUVEC. The effects of PM are probably mediated by its bioactive compound, quercetin-3-*O*-glucuronide. The findings may provide a rationale for the *in vivo* anti-atherosclerosis effect of PM, and support its potential use in atherosclerosis.

## 1. Introduction

Atherosclerotic cardiovascular disease is the main underlying cause of mortality throughout the world. Endothelial dysfunction is known as the precursor to atherosclerosis [1] and it can be detected in small resistance arteries much earlier before atherosclerotic plaque formation becomes visible on ultrasonography [2]. At the beginning of atherosclerosis development, an insult to the endothelial lining causes activation of the endothelial cells. Activated endothelium renders cellular adhesion molecules such as intercellular adhesion molecule-1 (ICAM-1) and vascular cell adhesion molecule-1 (VCAM-1). Cellular adhesion molecules enhance the adhesion of circulating monocytes to the activated endothelial cells. Subsequently, the monocytes infiltrate the endothelium and migrate into the subendothelial layer where they transform into macrophages which eventually form the foam cells in atherosclerotic plaque [3]. 

Pro-inflammatory cytokines such as tumor necrosis factor-α (TNF-α) have a significant role in endothelial dysfunction and atherosclerosis development [4]. TNF-α level was elevated in the plasma of patients with acute coronary syndrome [5], while deletion of TNF-α gene reduced the formation of atherosclerosis in mice [6]. TNF-α promotes atherogenesis by activating a transcription factor, nuclear factor kappaB (NF-κB). NF-κB activation upregulates cellular adhesion molecules expression, such as ICAM-1 and VCAM-1 [7]. For this reason, TNF-α has been widely used to induce vascular endothelial inflammation in various studies [8,9]. Since endothelial inflammation is a crucial step in atherogenesis [10,11,12], natural products that inhibit TNF-α-induced NF-κB activation and the consequent inflammation are the promising future therapies for atherosclerosis. 

*Persicaria minor* (Huds.) Opiz (syn. *Polygonum minus* Huds.) is a perennial herb that is commonly found in Southeast Asia, particularly in Malaysia, Indonesia, Thailand, and Vietnam. The herb is a member of the Polygonaceae family. This aromatic plant has been consumed as an ingredient in hot, spicy, and sour Malay delicacies [13]. Traditionally, *P. minor* is utilized as postnatal tonic and for dyspepsia and dandruff treatment. Acute and subchronic toxicity studies in female Sprague Dawley rats showed that *P. minor* is safe to consume up to a maximum tested dose of 2000 mg/kg [14]. *P. minor* is rich in phenolic and flavonoid compounds such as myricetin, quercetin [15], apigetrin, hyperoside, astragalin, miquelianin, isoquercetin, quercetin-3-*O*-glucuronide (Q3G), and quercitrin [16], which have been linked with its antioxidative and anti-inflammatory effects. 

*P. minor* has garnered wide attention due to its potential cardiovascular health benefits associated with its antioxidative and anti-inflammatory properties. The anti-inflammatory and antioxidative properties of *P. minor* are well investigated in *in vitro* and *in vivo* animal studies [17]. *P. minor* exhibits its antioxidative effect by directly scavenging superoxide anions [18,19,20], inhibiting lipid peroxidation [21,22], and promoting superoxide dismutase activity [17]. In comparison to the extensive literature on *P. minor’s* antioxidative effect, studies on its anti-inflammatory effect remain scarce. In a previous study, the ethanolic extract of *P. minor* leaves reduced inflammation by inhibiting cyclooxygenase (COX)-1, COX-2, and 5-lipoxygenase activities *in vitro*. Additionally, *P. minor* aqueous extract reduces carrageenan-induced paw edema in rats [23]. The ethanolic extract of *P. minor* leaves also protects the mouse aortic wall from the atherosclerotic changes induced by cadmium chloride exposure. Cadmium chloride is an environmental pollutant that stimulates reactive oxygen species formation and has been associated with endothelial dysfunction and atherosclerosis development [24]. 

Even though *P. minor* has demonstrated an anti-atherosclerosis effect, the mechanism underlying the anti-atherosclerosis effect of *P. minor* remains poorly understood. Therefore, this study investigated the ability of *P. minor* to attenuate endothelial activation and monocyte adherence to human umbilical vein endothelial cells (HUVEC), which are among the pivotal early steps in the pathogenesis of atherosclerosis. Furthermore, NF-κB activation was measured since it is the key transcription factor which modulates the expression of cellular adhesion molecules. A greater understanding of this aspect might provide a better insight on the prevention of endothelial activation and atherosclerosis. 

## 2. Materials and Methods

### 2.1. Standardized Aqueous Extract of P. minor Leaves Preparation

**Figure 1:**
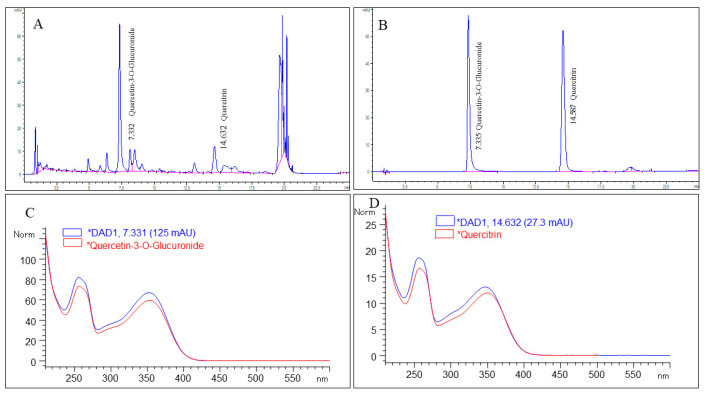
HPLC profile of standardized aqueous extract of *P. minor* leaves. (**A**) The major peaks correspond to quercetin-3-*O*-glucuronide at retention time (R*_t_*) 7.332 min and quercitrin at R*_t_* 14.632 min, respectively. (**B**) Quercetin-3-*O*-glucuronide and quercitrin standards eluted at R*_t_* 7.335 min and 14.597 min. (**C**) UV spectrum of quercetin-3-*O*-glucuronide with respect to its R*_t_*. (**D**) UV spectrum of quercitrin with respect to its R*_t_*.

*P. minor* leaves were supplied by Biotropics Malaysia Berhad and characterized by a plant taxonomist in Institute of Bioscience, Universiti Putra Malaysia (specimen voucher number: MFI 0112/19). The aqueous extract of *P. minor* leaves (PM) was optimized by high-performance liquid chromatography (HPLC) to have at least 0.59% Q3G and 0.27% quercitrin as bioactive compounds in accordance with Biotropics Malaysia Berhad’s in-house HPLC procedures [25]. Briefly, fresh *P. minor* leaves were oven-dried to a moisture content of less than 10%. The leaves were then cut into pieces and the extraction was carried out by soaking the leaves in water (1:20 *w*/*v*) and percolating for two cycles at 80 °C for 4 h. Subsequently, the extract was filtered, evaporated, and freeze-dried until it contained less than 8% water by weight. Then, the HPLC fingerprint of PM was obtained using Kinetex column 1.7 µm (C18, 2.1 × 150 mm). The mobile phase consisted of solvent X (0.10% formic acid in water) and Y (0.10% formic acid in acetonitrile) mixed following a linear gradient program of between 5–89% solvent X and 95–11% of solvent Y [25]. Peaks identified as Q3G and quercitrin at retention time (R*_t_*) 7.332 and 14.632 min, respectively, were validated by comparing their R*_t_* and UV spectrum with the standards (Figure 1).

### 2.2. HUVEC Isolation and Culture

This study was authorized by the Research Ethics Committee of Universiti Kebangsaan Malaysia Medical Centre (Study Code: UKM PPI/111/8/JEP-2019-671). Umbilical cords were obtained from consenting donors at the labor room of Hospital Canselor Tuanku Mukhriz, Malaysia. Informed consent was obtained from all the donors. HUVEC were derived from fresh human umbilical cords using collagenase digestion (Gibco-Invitrogen Corp., Grand Island, NY, USA) as previously reported [26]. HUVEC were identified by the characteristic cobblestone morphology and expression of CD31 and vonWillebrand factor by immunocytochemistry. The cells were cultivated in endothelial cell media (ScienCell Research Laboratories, San Diego, CA, USA) at 37 °C in a humidified environment containing 5% CO_2_ and 95% air. The medium was replaced every two days. For subsequent experiments, HUVEC were subcultured at 80% confluency until they reached passage three.

### 2.3. Determination of Cytotoxicity and Optimal Dose of PM on HUVEC Viability by 3-(4,5-Dimethylthiazol-2-YI)-2,5-Diphenyltetrazolium Bromide (MTT) Assay

The effect of PM on HUVEC viability as measured by MTT assay was used to determine the extract’s cytotoxicity. In a 96-well plate, 5 × 10^4^ HUVEC were seeded per well and incubated for 24 h to allow HUVEC to adhere. The cells were then exposed to PM for 24 h at varied concentrations between 10–500 µg/mL. Then, in each well, 20 µL of MTT solution was added. The supernatant was aspirated following 4 h of incubation, and the purple formazan crystals generated in the wells were dissolved in 200 µL of dimethyl sulfoxide. Using a multiplate reader, the absorbance of each well was measured at 570 nm. Since the first stage of MTT assay showed that PM up to the dose of 500 µg/mL was not cytotoxic to HUVEC, we proceeded with the second stage of MTT assay using a similar dose range to establish the optimal dose of PM for further experiments. For the second stage of MTT assay, HUVEC were pretreated with 10–500 µg/mL PM for 18 h, followed by induction with 30 ng/mL TNF-α (R&D Systems, Abingdon, UK) for 6 h. The TNF-α concentration of 30 ng/mL was selected since it was the first concentration that decreased the viability of HUVEC significantly [26]. The MTT assay was then conducted as described to evaluate HUVEC viability. 

### 2.4. HUVEC Treatment Protocol

For all experiments, HUVEC at passage 3 were cultured until reaching 80% confluency. Subsequently, the cells were assigned to five groups: (1) control, (2) treatment with 300 µg/mL PM for 24 h, (3) induction with 30 ng/mL TNF-α for 6 h to stimulate inflammation, (4) pretreatment with 300 µg/mL PM for 18 h followed by 30 ng/mL TNF-α for 6 h, and (5) pretreatment with 1.35 µg/mL Q3G for 18 h followed by 30 ng/mL TNF-α for 6 h. The concentration of TNF-α used was based on its inhibitory effect on HUVEC viability from a previous study [26]. Based on the results of the MTT assay described in Section 2.3, the optimal dose of PM (300 µg/mL) was chosen. As PM contained 0.59% Q3G, the dose of Q3G (1.35 µg/mL) was calculated based on the concentration of Q3G present in 300 µg/mL PM.

### 2.5. Measurement of ICAM-1 and VCAM-1 mRNA Expressions Using Quantitative Real-Time Polymerase Chain Reaction (qPCR)

Total RNA was isolated from HUVEC using TRI reagent (Molecular Research Centre, Cincinnati, OH, USA) based on the reagent’s protocol. The total RNA was converted to cDNA with SuperScript^®^ III First-Strand Synthesis SuperMix for qPCR kit (Invitrogen, Carlsbad, CA, USA). qPCR was done according to the method published by Ugusman et al. [27] using QuantiNova SYBR^®^ Green PCR Kit (Qiagen, Germantown, MD, USA). The PCR reaction consisted of cDNA, SYBR^®^ Green PCR Master Mix, forward and reverse primers, and RNAse- and DNAse-free distilled water. The sequence of the forward and reverse primers used were as follows: GAPDH (GenBank accession no: NM_002046), forward: 5′- TCCCTGAGCTGAACGGGAAG-3′, reverse: 5′- GGAGGAGTGGGTGTCGCTGT-3′; ICAM-1 (GenBank accession no: NM_000201), forward: 5′- CAGTCACCTATGGCAACGACT-3′, reverse: 5′- CTCTGGCTTCGTCAGAATCAC-3′; VCAM-1 (GenBank accession no: NM_ 001078), forward: 5′- AGTTGAAGGATGCGGGAGTAT-3′, reverse: 5′- GGATGCAAAATAGAGCACGAG-3′. Using CFX96^TM^ Real-Time PCR Detection system (Biorad, USA), PCR was conducted with an initial denaturing step at 95 °C for 3 min; 40 cycles of 61 °C for 30 s, 95 °C for 1 min, 55 °C for 1 min, 70 cycles of 60 °C for 10 s; and ended by a cooling step at 4 °C. Each analysis was carried out in duplicates. The melting curves were analyzed to confirm the specificity of the reaction. The threshold cycle (C_T_) values were obtained, and the mRNA expression of ICAM-1 and VCAM-1 was determined as follows:

Relative mRNA expression = 2^−∆∆CT^

∆∆C_T_ = C_T_ GAPDH − C_T_ gene of interest

### 2.6. Measurement of Protein Levels of ICAM-I and VCAM-1 in HUVEC Using Enzyme-Linked Immunosorbent Assay (ELISA)

Human VCAM-1 and ICAM-1 ELISA kits (Qayee-Bio, China) were used to assess VCAM-1 and ICAM-1 protein levels in HUVEC. The assay was conducted in accordance with the kit’s protocol. The culture medium was removed and HUVEC were washed once with phosphate-buffered saline (PBS). Then, lysis buffer was added to the cells prior to scraping and collecting the cell lysates in microcentrifuge tubes. The cell lysates were put on ice for 30 min. Following this step, the cell lysates were centrifuged for 10 min at 8000 rpm. Supernatants containing the cell extract were collected and used as samples for ELISA. The samples were added into a plate coated with human anti-ICAM-1 and -VCAM-1. Next, horseradish peroxidase (HRP)-conjugated avidin and tetramethylbenzidine (TMB) substrate were pipetted into the plate prior to the stop solution. At 450 nm, the plate’s optical density was measured. Total protein levels in HUVEC samples were measured using the Bradford technique [28]. The protein levels of ICAM-1 and VCAM-1 in HUVEC were normalized to the amount of total protein in the samples. 

### 2.7. Measurement of Monocyte Adhesion to HUVEC

HUVEC were washed with PBS according to the procedure outlined in Section 2.4. Subsequently, 1 × 10^5^ U937 monocytes (ATCC^®^ CRL-1593.2^TM^) were added to HUVEC and incubated at 37 °C for 30 min. Following the 30 min incubation period, the unattached U937 monocytes were washed away using PBS. Under an inverted light microscope, the U937 cells that adhered to the surface of HUVEC were recognized and calculated [29]. 

### 2.8. Measurement of NF-kB p65 Levels in HUVEC

Human NF-κB p65 subunit ELISA kit (Elabscience, Houston, TX, USA) was utilized to determine NF-κB p65 concentration in the nuclear extract of HUVEC. The procedures were done in conformity with the manufacturer’s guidelines. Nuclear extract of HUVEC was prepared by sonicating HUVEC samples, followed by diluting the cells in lysis buffer and centrifuging for 20 min at 8000 rpm. The samples were pipetted into NF-κB p65-coated plate and incubated for 2 h. Then, HRP-conjugated streptavidin, TMB substrate and stop solution were added. The plate absorbance was recorded at 450 nm wavelength. The level of NF-κB p65 in HUVEC nuclear extract was normalized with the sample’s total protein content. 

### 2.9. Data Analysis

The results were analyzed using the GraphPad Prism8 software (GraphPad, San Diego, CA, USA). Kolmogorov–Smirnov test was performed to assess the data normality. The data are presented as mean ± standard error for mean (SEM). One-way ANOVA with post-hoc Tukey test was used to assess the statistical difference between the groups. *p* values < 0.05 were taken as statistically significant.

## 3. Results

### 3.1. PM Improved the Viability of HUVEC Exposed to TNF-α

**Figure 2:**
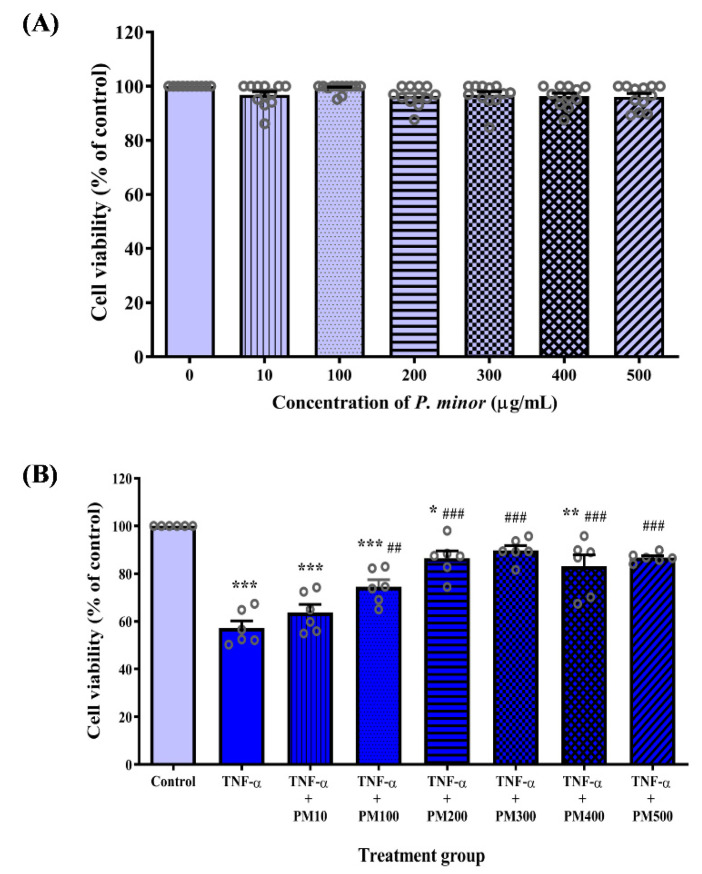
Effect of standardized aqueous extract of *P. minor* leaves (PM) on the viability of tumor necrosis factor (TNF)-α-induced human umbilical vein endothelial cells (HUVEC). (**A**) Toxicity of PM in HUVEC was determined by 3-(4,5-dimethylthiazol-2-yl)-2,5-diphenyltetrazolium bromide (MTT) assay following treatment with 10–500 μg/mL PM for 24 h. (**B**) HUVEC were pretreated with 10–500 μg/mL PM for 18 h, followed by exposure to 30 ng/mL TNF-α for 6 h, then the cell viability was measured. Values are shown as the mean ± SEM, *n* = 6–12 (* *p* < 0.05; ** *p* < 0.01; *** *p* < 0.001 vs. control. ^##^ *p* < 0.01; ^###^ *p* < 0.001 vs. TNF-α).

Treatment with various concentrations of PM ranging from 10 to 500 µg/mL did not have a cytotoxic effect on HUVEC as the cell viability was above 90% for all concentrations tested (Figure 2A). In the second stage of MTT assay (Figure 2B), HUVEC stimulated with TNF-α had significantly reduced cell viability compared with the control cells (*p* < 0.001). Pretreatment of TNF-α-induced HUVEC with PM (100–500 µg/mL) dose-dependently increased cell viability (*p* < 0.001–0.01). PM at a concentration of 300 µg/mL was the first dose that increased TNF-α-induced HUVEC viability to a level comparable with the control group. Therefore, 300 µg/mL PM was chosen as the optimal dose for subsequent experiments.

### 3.2. Effect of PM on ICAM-1 and VCAM-1 mRNA Expression and Protein Levels in HUVEC

**Figure 3:**
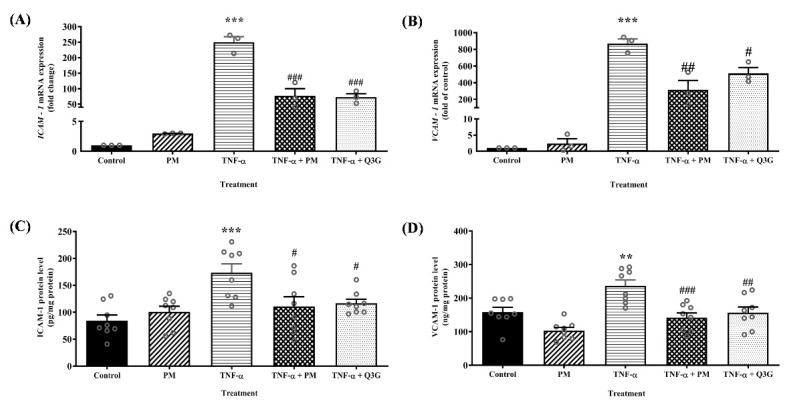
(**A**) ICAM-1 mRNA expression. (**B**) VCAM-1 mRNA expression. (**C**) ICAM-1 protein level. (**D**) VCAM-1 protein level in human umbilical vein endothelial cells (HUVEC). Values are expressed as mean ± SEM, *n* = 3–8 (** *p* < 0.01; *** *p* < 0.001 vs. control. ^#^ *p* < 0.05; ^##^ *p* < 0.01; ^###^ *p* < 0.001 vs. TNF-α). ICAM-1, intercellular adhesion molecule-1; PM, standardized aqueous extract of *P. minor* leaves; Q3G, quercetin-3-*O*-glucuronide; TNF-α, tumor necrosis factor-α; VCAM-1, vascular cell adhesion molecule-1.

Treatment with PM alone had no significant effect on ICAM-1 and VCAM-1 mRNA expression and protein levels compared to the control group (Figure 3). In the TNF-α group, mRNA expression of ICAM-1 and VCAM-1 was upregulated by 250-fold (*p* < 0.001) and 900-fold (*p* < 0.001), respectively. Consequently, HUVEC treated with TNF-α also showed higher protein levels of ICAM-1 (*p* < 0.001) and VCAM-1 (*p* < 0.01) compared to the control group. Treatment of TNF-α-induced HUVEC with PM attenuated ICAM-1 mRNA expression (*p* < 0.001) and protein levels (*p* < 0.05), as well as VCAM-1 mRNA expression (*p* < 0.01) and protein levels (*p* < 0.001). Similarly, treatment with Q3G reduced ICAM-1 mRNA expression (*p* < 0.001) and protein levels (*p* < 0.05), as well as VCAM-1 mRNA expression (*p* < 0.05) and protein levels (*p* < 0.01). There was no significant difference in the mRNA expression and protein levels of ICAM-1 and VCAM-1 between the PM and Q3G group.

### 3.3. Effect of PM on U937 Monocyte Adhesion to HUVEC

**Figure 4:**
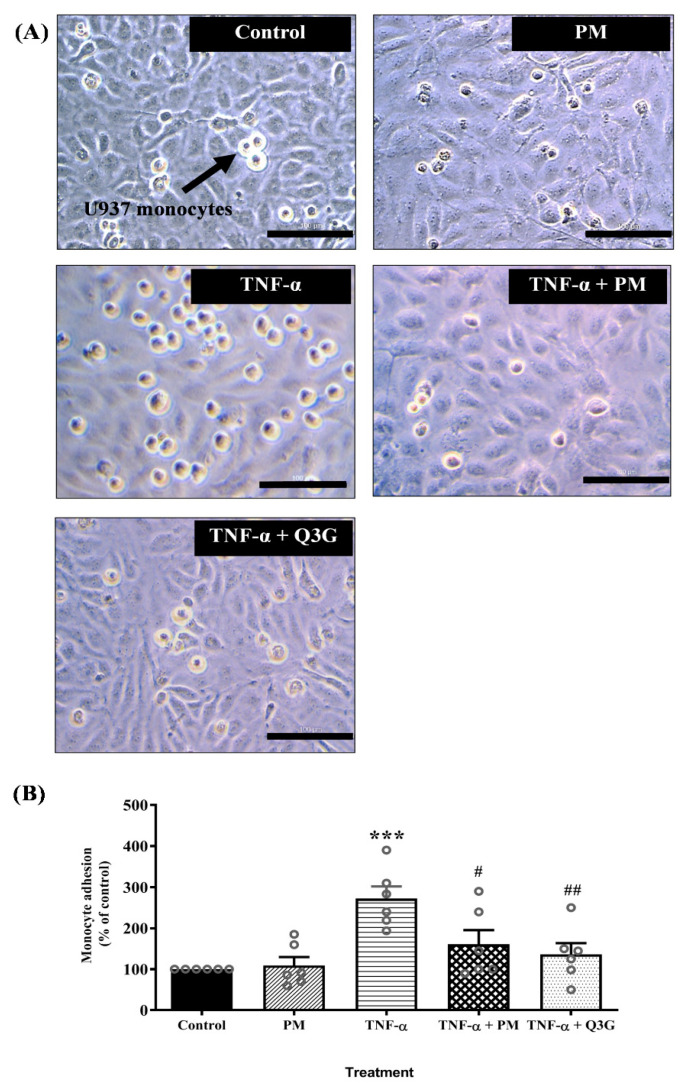
(**A**) Microscopic examination showing reduced number of U397 monocytes that adhered to tumor necrosis factor (TNF)-α-induced human umbilical vein endothelial cells (HUVEC) pretreated with standardized aqueous extract of *P. minor* leaves (PM) and quercetin-3-*O*-glucuronide (Q3G) (magnification: ×40, scale bar: 100 µM). (**B**) Quantitative analysis of monocyte adhesion to HUVEC. Values are expressed as mean ± SEM, *n* = 6 (*** *p* < 0.001 vs. control. ^#^ *p* < 0.05; ^##^ *p* < 0.01 vs. TNF-α).

Microscopic examination showed that exposure to TNF-α increased monocyte adhesion to HUVEC and the effect was reduced when HUVEC were treated with PM and Q3G (Figure 4A). Quantitative analysis confirmed that PM alone did not cause any significant change in adhesion of monocytes to HUVEC compared to the control group (Figure 4B). Exposure to TNF-α increased monocyte adhesion to HUVEC compared to the control group (*p* < 0.001). Monocyte adhesion decreased when TNF-α-induced HUVEC were treated with PM (*p* < 0.05) and Q3G (*p* < 0.01) compared to the TNF-α group. There was no significant difference in adhesion of monocytes to HUVEC between the PM and Q3G group.

### 3.4. Effect of PM on NF-κB p65 Level in HUVEC Nuclear Extract

**Figure 5:**
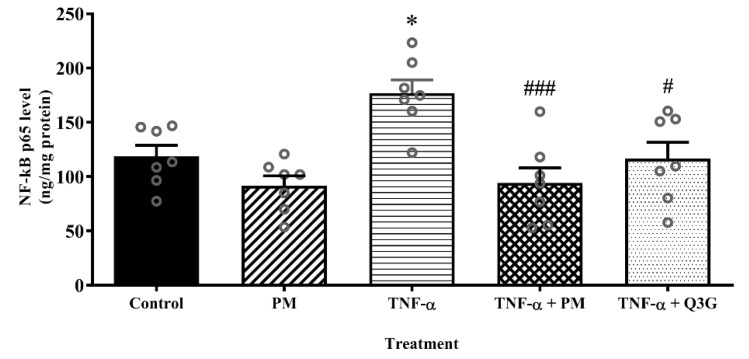
Nuclear factor kappaB (NF-κB) p65 levels in the nuclear extract of human umbilical vein endothelial cells (HUVEC). Values are expressed as mean ± SEM, *n* = 7 (* *p* < 0.05 vs. control. ^#^ *p* < 0.05; ^###^ *p* < 0.001 vs. TNF-α). PM, standardized aqueous extract of *P. minor* leaves; Q3G, quercetin-3-*O*-glucuronide; TNF-α, tumor necrosis factor-α.

The nuclear extract of HUVEC treated with PM alone did not demonstrate any significant difference in the level of NF-κB p65 when compared to the control (Figure 5). However, stimulation of HUVEC with TNF-α led to a higher level of NF-κB p65 compared to the control (*p* < 0.05). When TNF-α-induced HUVEC were treated with PM, the level of NF-κB p65 was lower compared to the TNF-α group (*p* < 0.001). Similarly, treatment of TNF-α-induced HUVEC with Q3G also decreased the NF-κB p65 level (*p* < 0.05). The levels of NF-κB p65 in PM and Q3G groups were not statistically different.

## 4. Discussion

Using TNF-α-induced HUVEC as the *in vitro* model of vascular inflammation and atherogenesis, in this study, we demonstrated that PM and its bioactive compound, Q3G, have a protective effect against endothelial activation in atherogenesis by inhibiting TNF-α-induced activation of NF-κB, expression of adhesion molecule and adhesion of monocytes to HUVEC. The mRNA expression and protein levels of VCAM-1 and ICAM-1 were remarkably increased in TNF-α-induced HUVEC. Consequently, monocyte adhesion to HUVEC also increased in response to TNF-α. These results were congruent with a prior study in which TNF-α stimulated VCAM-1 and ICAM-1 expression, which enhanced monocyte adherence to HUVEC [30]. Monocyte-endothelial cell adhesion is one of the key inflammatory steps in the development of atherosclerosis. In the early stage of atherogenesis, upregulation of cellular adhesion molecules at the endothelial lining is responsible for promoting the adherence of circulating monocytes to the endothelial surface [31]. This is followed by the migration of the monocytes to the subendothelial layer before they transform into foam cells and macrophages, which are parts of an atherosclerotic plaque [3,32]. 

We demonstrated that TNF-α-induced VCAM-1 and ICAM-1 mRNA expression and protein levels were attenuated by PM. The decrease in the expression of cellular adhesion molecules subsequently hindered monocyte adherence to HUVEC. These effects of PM are most likely contributed by Q3G as treatment with Q3G also produced similar, comparable effects. The findings are consistent with an earlier study that showed Q3G inhibits ICAM-1 and VCAM-1 expression in HUVEC stimulated with TNF-α [33]. In contrast, Q3G has no suppressive effect on ICAM-1 expression in TNF-α-induced HAEC and human vascular smooth muscle cells [34,35]. Although cultured endothelial cells are commonly used to represent the vascular endothelium, endothelial cells from different vascular beds respond in distinct ways to various cytokines like TNF-α [36].

To examine the influence of PM in the transcriptional control of VCAM-1 and ICAM-1 expression, we measured the amount of NF-κB p65 in the nuclear extract of TNF-α-stimulated HUVEC. The results indicated that when HUVEC was induced with TNF-α, the amount of NF-κB p65 surged in the nuclear extract. TNF-α stimulates phosphorylation of IkB, an inhibitory protein that keeps NF-κB inactive in the cell cytoplasm. Following the breakdown of IkB, NF-κB moves from the cytoplasm to the nucleus and binds to specific sequences of DNA, hence activating the target gene transcription. NF-κB p65 subunit is required to commence target gene transcription [37,38]. NF-κB binding to the promoters of VCAM-1 and ICAM-1 enhances their gene expression, which eventually stimulates monocyte adherence to the endothelium [39]. 

As treatment with PM and Q3G reduced NF-κB p65 levels, the decline in ICAM-1 and VCAM-1 levels in TNF-α-induced HUVEC with PM and Q3G treatment was partially mediated by inhibition of NF-κB activation. Previous study showed that Q3G reduces inflammation and NF-κB activation in HUVEC by inhibiting p65 phosphorylation [40]. In addition to NF-κB, TNF-α also activates other transcription factors, including activator protein-1 (AP-1) via the mitogen-activated protein kinase (MAPK) signaling pathway. AP-1 is involved in the stimulation of VCAM-1 and ICAM-1 expression [41]. However, the influence of PM on AP-1 activity is not evaluated in this study, hence the need for further investigation.

## 5. Conclusions

*P. minor* attenuates TNF-α-induced expression of cellular adhesion molecules and adherence of monocytes to endothelial cells. Suppression of the adhesion of monocytes to endothelial cells by *P. minor* is potentially achieved via a reduction in NF-κB activation. The positive effects of *P. minor* are most probably mediated by its bioactive compound, Q3G. These findings suggest that *P. minor* can be a protective agent against endothelial activation, which is related to the early phase of atherosclerosis. However, apart from NF-κB, this study does not assess the involvement of other transcription factors such as AP-1 that may also modulate the cellular adhesion molecules expression through the MAPK signal transduction pathway. Studies that evaluate the role of PM in modulating inflammation via the MAPK pathway as well as *in vivo* studies involving atherosclerosis animal models are required to further support these *in vitro* study findings.

## Data Availability

The data used to support the findings of this study are included in the article.

## References

[1] Soehnlein O., Libby P. (2021). Targeting Inflammation in Atherosclerosis—from Experimental Insights to the Clinic. Nat. Rev. Drug Discov..

[2] Davignon J., Ganz P. (2004). Role of Endothelial Dysfunction in Atherosclerosis. Circulation.

[3] Čejková S., Králová-Lesná I., Poledne R. (2016). Monocyte Adhesion to the Endothelium Is an Initial Stage of Atherosclerosis Development. Cor Vasa.

[4] Botts S.R., Fish J.E., Howe K.L. (2021). Dysfunctional Vascular Endothelium as a Driver of Atherosclerosis: Emerging Insights Into Pathogenesis and Treatment. Front. Pharmacol..

[5] Al Shahi H., Shimada K., Miyauchi K., Yoshihara T., Sai E., Shiozawa T., Naito R., Aikawa T., Ouchi S., Kadoguchi T. (2015). Elevated Circulating Levels of Inflammatory Markers in Patients with Acute Coronary Syndrome. Int. J. Vasc. Med..

[6] Ohta H., Wada H., Niwa T., Kirii H., Iwamoto N., Fujii H., Saito K., Sekikawa K., Seishima M. (2005). Disruption of Tumor Necrosis Factor-α Gene Diminishes the Development of Atherosclerosis in ApoE-Deficient Mice. Atherosclerosis.

[7] Galkina E., Ley K. (2007). Vascular Adhesion Molecules in Atherosclerosis. Arterioscler. Thromb. Vasc. Biol..

[8] Habas K., Shang L. (2018). Alterations in Intercellular Adhesion Molecule 1 (ICAM-1) and Vascular Cell Adhesion Molecule 1 (VCAM-1) in Human Endothelial Cells. Tissue Cell.

[9] Chen X., Li H., Wang Z., Zhou Q., Chen S., Yang B., Yin D., He H., He M. (2020). Quercetin Protects the Vascular Endothelium against Iron Overload Damages via ROS/ADMA/DDAHⅡ/ENOS/NO Pathway. Eur. J. Pharmacol..

[10] Toma L., Sanda G.M., Niculescu L.S., Deleanu M., Sima A.V., Stancu C.S. (2020). Phenolic Compounds Exerting Lipid-Regulatory, Anti-Inflammatory and Epigenetic Effects as Complementary Treatments in Cardiovascular Diseases. Biomolecules.

[11] Chen C., Luo F., Liu X., Lu L., Xu H., Yang Q., Xue J., Shi L., Li J., Zhang A. (2017). NF-KB-Regulated Exosomal MiR-155 Promotes the Inflammation Associated with Arsenite Carcinogenesis. Cancer Lett..

[12] Zinatizadeh M.R., Schock B., Chalbatani G.M., Zarandi P.K., Jalali S.A., Miri S.R. (2021). The Nuclear Factor Kappa B (NF-KB) Signaling in Cancer Development and Immune Diseases. Genes Dis..

[13] Burkill I.H. (1936). A Dictionary of the Economic Products of the Malay Peninsula. Nature.

[14] Christapher P.V., Parasuraman S., Asmawi M.Z., Murugaiyah V. (2017). Acute and Subchronic Toxicity Studies of Methanol Extract of Polygonum Minus Leaves in Sprague Dawley Rats. Regul. Toxicol. Pharmacol..

[15] Christapher P., Vikneswaran M., Xin T., Yuan G., Parasuraman S., Leng L., Kiun C., Fu N. (2015). Evaluation of Analgesic, Anti-Inflammatory, Antipyretic and Antiulcer Effect of Aqueous and Methanol Extracts of Leaves of Polygonum Minus Huds. (Polygonaceae) in Rodents. Arch. Med. Health Sci..

[16] Abdullah M.Z., Mohd Ali J., Abolmaesoomi M., Abdul-Rahman P.S., Hashim O.H. (2017). Anti-Proliferative, in Vitro Antioxidant, and Cellular Antioxidant Activities of the Leaf Extracts from Polygonum Minus Huds: Effects of Solvent Polarity. Int. J. Food Prop..

[17] Hamid A.A., Aminuddin A., Yunus M.H.M., Murthy J.K., Hui C.K., Ugusman A. (2020). Antioxidative and Anti-Inflammatory Activities of Polygonum Minus: A Review of Literature. Rev. Cardiovasc. Med..

[18] Sumazian Y., Syahida A., Hakiman M., Maziah M. (2010). Antioxidant Activities, Flavonoids, Ascorbic Acid and Phenolic Contents of Malaysian Vegetables. J. Med. Plants Res..

[19] Maizura M., Aminah A., Aida W.M.W. (2011). Total Phenolic Content and Antioxidant Activity of Kesum (*Polygonum Minus*), Ginger (*Zingiber Officinale*) and Turmeric (*Curcuma Longa*) Extract. Int. Food Res. J..

[20] Wan Yahaya W.A., Abu Yazid N., Mohd Azman N.A., Almajano M.P. (2019). Antioxidant Activities and Total Phenolic Content of Malaysian Herbs as Components of Active Packaging Film in Beef Patties. Antioxidants.

[21] Nurul H., Ruzita A., Aronal A.P. (2010). The Antioxidant Effects of Cosmos Caudatus and Polygonum Minus in Refrigerated Duck Meatballs. Int. Food Res. J..

[22] Saputri F.C., Jantan I. (2011). Effects of Selected Medicinal Plants on Human Low-Density Lipoprotein Oxidation, 2, 2-Diphenyl-1-Picrylhydrazyl (DPPH) Radicals and Human Platelet Aggregation. J. Med. Plant Res..

[23] George A., Chinnappan S., Chintamaneni M., Kotak C.V., Choudhary Y., Kueper T., Radhakrishnan A.K. (2014). Anti-Inflammatory Effects of Polygonum Minus (Huds) Extract (Lineminus^TM^) in in-Vitro Enzyme Assays and Carrageenan Induced Paw Edema. BMC Complement. Altern. Med..

[24] Kusumaningrum P.A., Yustinasari L.R., Sahrial I., Hamid, Sudjarwo S.A., Kuncoro Puguh Santoso C.A. (2019). Effect of Polygonum Minus (KESUM) leaves ethanolic extract on histopathological changes on the wall aorta of mice (Mus Musculus) induced by cadmium chloride antioxidant. J. Basic Med. Vet..

[25] George A., Ng C.P., O’Callaghan M., Jensen G.S., Wong H.J. (2014). In Vitro and Ex-Vivo Cellular Antioxidant Protection and Cognitive Enhancing Effects of an Extract of Polygonum Minus Huds (Lineminus^TM^) Demonstrated in a Barnes Maze Animal Model for Memory and Learning. BMC Complement. Altern. Med..

[26] Sundar U.M., Ugusman A., Chua H.K., Latip J., Aminuddin A. (2019). Piper Sarmentosum Promotes Endothelial Nitric Oxide Production by Reducing Asymmetric Dimethylarginine in Tumor Necrosis Factor-α-Induced Human Umbilical Vein Endothelial Cells. Front. Pharmacol..

[27] Ugusman A., Zakaria Z., Hui C.K., Megat Mohd Nordin N.A. (2011). Piper Sarmentosum Inhibits ICAM-1 and Nox4 Gene Expression in Oxidative Stress-Induced Human Umbilical Vein Endothelial Cells. BMC Complement. Altern. Med..

[28] Bradford M.M. (1976). A Rapid and Sensitive Method for the Quantitation of Microgram Quantities of Protein Utilizing the Principle of Protein-Dye Binding. Anal. Biochem..

[29] Chang C.C., Chu C.F., Wang C.N., Wu H.T., Bi K.W., Pang J.H.S., Huang S.T. (2014). The Anti-Atherosclerotic Effect of Tanshinone IIA Is Associated with the Inhibition of TNF-α-Induced VCAM-1, ICAM-1 and CX3CL1 Expression. Phytomedicine.

[30] Ismail S.M., Sundar U.M., Hui C.K., Aminuddin A., Ugusman A. (2018). Piper Sarmentosum Attenuates TNF-α-Induced VCAM-1 and ICAM-1 Expression in Human Umbilical Vein Endothelial Cells. J. Taibah Univ. Med. Sci..

[31] Cook-Mills J.M., Marchese M.E., Abdala-Valencia H. (2011). Vascular Cell Adhesion Molecule-1 Expression and Signaling during Disease: Regulation by Reactive Oxygen Species and Antioxidants. Antioxid. Redox Signal..

[32] Maguire E.M., Pearce S.W.A., Xiao Q. (2019). Foam Cell Formation: A New Target for Fighting Atherosclerosis and Cardiovascular Disease. Vascul. Pharmacol..

[33] Tribolo S., Lodi F., Connor C., Suri S., Wilson V.G., Taylor M.A., Needs P.W., Kroon P.A., Hughes D.A. (2008). Comparative Effects of Quercetin and Its Predominant Human Metabolites on Adhesion Molecule Expression in Activated Human Vascular Endothelial Cells. Atherosclerosis.

[34] Mochizuki M., Kajiya K., Terao J., Kaji K., Kumazawa S., Nakayama T., Shimoi K. (2004). Effect of Quercetin Conjugates on Vascular Permeability and Expression of Adhesion Molecules. BioFactors.

[35] Winterbone M.S., Tribolo S., Needs P.W., Kroon P.A., Hughes D.A. (2009). Physiologically Relevant Metabolites of Quercetin Have No Effect on Adhesion Molecule or Chemokine Expression in Human Vascular Smooth Muscle Cells. Atherosclerosis.

[36] Krüger-Genge A., Blocki A., Franke R.P., Jung F. (2019). Vascular Endothelial Cell Biology: An Update. Int. J. Mol. Sci..

[37] Bhatt D., Ghosh S. (2014). Regulation of the NF-ΚB-Mediated Transcription of Inflammatory Genes. Front. Immunol..

[38] Milstone D.S., Ilyama M., Chen M., O’Donnell P., Davis V.M., Plutzky J., Brown J.D., Haldar S.M., Siu A., Lau A.C. (2015). Differential Role of an NF-ΚB Transcriptional Response Element in Endothelial versus Intimal Cell VCAM-1 Expression. Circ. Res..

[39] Ernst O., Vayttaden S.J., Fraser I.D.C. (2018). Measurement of NF-ΚB Activation in TLR-Activated Macrophages. Methods Mol. Biol..

[40] Guo X.D., Zhang D.Y., Gao X.J., Parry J., Liu K., Liu B.L., Wang M. (2013). Quercetin and Quercetin-3-O-Glucuronide Are Equally Effective in Ameliorating Endothelial Insulin Resistance through Inhibition of Reactive Oxygen Species-Associated Inflammation. Mol. Nutr. Food Res..

[41] Chen T., Zhang X., Zhu G., Liu H., Chen J., Wang Y., He X. (2020). Quercetin Inhibits TNF-α Induced HUVECs Apoptosis and Inflammation via Downregulating NF-KB and AP-1 Signaling Pathway in Vitro. Medicine.

